# Sulforaphane inhibits cancer stem-like cell properties and cisplatin resistance through miR-214-mediated downregulation of c-MYC in non-small cell lung cancer

**DOI:** 10.18632/oncotarget.14512

**Published:** 2017-01-05

**Authors:** Qian-Qian Li, You-Ke Xie, Yue Wu, Lin-Lin Li, Ying Liu, Xiao-Bo Miao, Qiu-Zhen Liu, Kai-Tai Yao, Guang-Hui Xiao

**Affiliations:** ^1^ Cancer Institute, Southern Medical University, Guangzhou 510515, China

**Keywords:** sulforaphane, c-MYC, miR-214, cisplatin, lung cancer

## Abstract

We herein report that sulforaphane (SFN), a potent anti-cancer and well-tolerated dietary compound, inhibits cancer stem-like cell (CSC) properties and enhances therapeutic efficacy of cisplatin in human non-small cell lung cancer (NSCLC). SFN exerted these functions through upregulation of miR-214, which in turn targets the coding region of *c-MYC*. This finding was further corroborated by our observations that plasmid or lentiviral vector-mediated expression of 3'UTR-less c-MYC cDNA and cisplatin- or doxorubicin-induced endogenous c-MYC accumulation was similarly suppressed by either SFN or miR-214. Further, we showed that the reported inhibitory effects of SFN on β-catenin are also mediated by miR-214. SFN/miR-214 signaling inhibited CSC properties and enhanced the cytotoxicity of chemotherapeutic drugs *in vitro*. Experiments with nude mice carrying xenograft tumors showed that SFN sensitized NSCLC cells to cisplatin's efficacy, which is accompanied by inhibition of cisplatin-induced c-MYC accumulation in tumor tissues. Our results provided strong evidence and mechanisms to support consideration of SFN or synthetic derivatives as a therapeutic agent in combination with cisplatin for the treatment of patients with NSCLC and, potentially, other types of c-MYC-addicted tumors.

## INTRODUCTION

Sulforaphane (SFN) is a natural isothiocyanate found in broccoli and other cruciferous vegetables and is one of the most effective chemopreventive agents against cancer [1]. The molecular targets and mechanisms of action of SFN have been extensively studied during the past two decades. Early research suggested that SFN affects tumorigenesis and tumor development through blocking the initiation stage of cancer by inhibiting the phase 1 metabolism enzymes and inducing phase 2 metabolism enzymes [2]. Subsequent studies disclosed that SFN can modulate diverse cellular activities, such as inhibiting cell proliferation, inducing apoptosis, and suppressing angiogenesis and metastasis [2, 3]. Recently, SFN was demonstrated to target cancer stem-like cells (CSCs), also called tumor-initiating cells, by suppressing NF-kappaB-induced antiapoptosis [4] or breast CSCs by downregulating the Wnt/β-catenin pathway [5].

Proto-oncogene *c-Myc*, encoding one of the most important transcription factors, plays a pivotal role in tumor initiation and progression [6, 7]. c-Myc regulates hundreds of disparate target genes that participate numerous biological effects, such as cell proliferation, apoptosis, differentiation, and stem cell self-renewal [8–10]. c-Myc is one of the four factors used in reprogramming somatic cells to induce pluripotent stem (iPS) cells [11] and is implicated in maintaining CSCs [12, 13]. Recently, a high-resolution analysis of somatic copy-number alterations (SCNAs) from 3131 cancer specimens revealed that amplification of the human *c-MYC* gene occurs in most types of malignancies, underscoring the critical role of c-MYC in tumor pathogenesis [14].

Non-small cell lung cancer (NSCLC) represents approximately 80% of all lung cancers and is the leading cause of cancer-related mortality worldwide [15, 16]. Although the initial chemotherapy is successful, intrinsic or acquired drug resistance leads to poor prognosis and disease relapse. Upregulation of *c-MYC* expression is frequently detected in NSCLC and is correlated with aggressive clinicopathological features and reduced disease-free survival [17–19]. In the present study, we show that SFN potently inhibited c-MYC expression through upregulating miR-214. We further investigated the functional impact of SFN/miR-214/c-MYC signaling on CSC properties and chemoresistance. Our results support further evaluation of SFN or pharmaceutical derivatives as a therapeutic agent for the treatment of NSCLC.

## RESULTS

### SFN inhibits cell viability, induces apoptosis, and represses cancer stem-like cell properties of NSCLC

We firstly evaluated effects of SFN on cell viability in a normal lung bronchial epithelium cell line BEAS-2B and three human NSCLC cell lines, H460, H1299 and A549. Compared to untreated cells, treatment with SFN markedly inhibited NSCLC cell viability, with an IC_50_ of 12, 8, and 10 μmol/L for H460, H1299 and A549, respectively. In contrast, BEAS-2B cells were significantly less sensitive to SFN treatment with an IC_50_ of 25.9 μmol/L (Supplementary Figure 1A). The effect of SFN on DNA synthesis was measured with a 5-ethynyl-2'-deoxyuridine (EdU) incorporation assay. SFN at 10 μmol/L decreased the percentage of EdU-positive cells in the three NSCLC lines, implying reduction of cells in S phase (Supplementary Figure 1B). The ability of SFN to induce apoptosis was assessed by using flow cytometric analysis with propidium iodide and Annexin V double staining. SFN significantly induced apoptosis in each of the three lines (Supplementary Figure 1C). These results are consistent with previous reports that SFN inhibited proliferation and induced apoptosis of NSCLC cells [20, 21].

Tumor spheroids propagated in defined condition were enriched for cells with cancer stem cell-like characteristics and recapitulate the phenotype and genotype of primary tumors [22]. Cells were cultured in serum-free medium containing bFGF and EGF plus SFN (5 μmol/L). Compared with vehicle treated cells, SFN significantly reduced the number of spheroids by 85%, 78%, and 79% for H460, H1299, and A549 cells, respectively (Supplementary Figure 2A).

Previous studies have shown that CD133+ cells exhibit self-renewal and tumor-initiating abilities in NSCLC [23]. We examined if SFN could suppress CD133+ population in H460 cells that have a higher CD133+ fraction (2~3%) than the other two cell lines. Flow cytometric analysis with a CD133 antibody revealed that SFN at 5 or 10 μmol/L markedly decreased the proportion of CD133+ cells by 43% and 87%, respectively (Supplementary Figure 2B).

**Figure 1:**
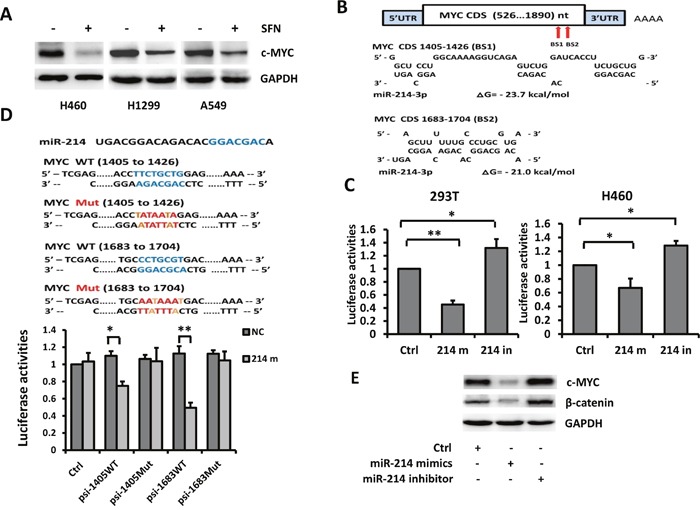
*c-MYC* is a direct target of miR-214 **A**. SFN downregulated the expression of c-MYC. H460, H1299 and A549 cells were treated with SFN (10 μmol/L) for 24 hours and subjected to Western blot analysis with indicated antibodies. **B**. Computational analyses with RNA22 and RNAhybrid algorithms predicted two miR-214 binding sites within the *c-MYC* CDS. Sequence alignments of miR-214 and the *c-MYC* are shown. **C**. Luciferase assays on 293T and H460 cells. Cells were co-transfected with a luciferase reporter containing the full length *c-MYC* CDS (psi-c-MYC-CDS) with NC-mimic control, miR-214 mimic, or miR-214 inhibitor. Luciferase activities were measured 48 hours after transfection. miR-214 mimic markedly suppressed luciferase activity and miR-214 inhibitor elevated luciferase activity. Columns, mean (n =3); bars, SD; * *p* < 0.01; **p < 0.001. **D**. H460 cells were co-transfected with a luciferase reporter containing a wild type or mutated miR-214 binding site (pWT1405, pMut1405, pWT1683, or pMut1683) together with NC or miR-214 mimic. Luciferase activities were measured 48 hours after transfection. Columns, mean (n =3); bars, SD; *, *p* < 0.05; **, p < 0.01. **E**. miR-214 downregulated endogenous c-MYC and β-catenin protein levels.

The potent anti-cancer and anti-CSC activity of SFN observed in above experiments prompted us to ask whether these effects of SFN are associated with inhibition of any CSC-related factors in NSCLC cells. To test this, H460, H1299, and A549 cells were treated with 10 μmol/L SFN followed by Western blot analyses. We found that c-MYC protein was moderately expressed in untreated cells and substantially down-regulated by SFN in each of the cell lines (Figure 1A).

### Identification of SFN-modulated miRNAs in NSCLC

We sought to elucidate the mechanism by which SFN regulates *c-MYC* expression. Since miRNAs are master regulators of various biological processes, we asked that whether SFN might regulate *c-MYC* expression via miRNA. For this purpose, H460 cells were treated with vehicle or SFN followed by TaqMan real-time PCR microRNA assays. Comparison of the miRNA expression profiles between the control and SFN treated samples revealed a number of miRNA including miR-214, miR-145, miR-199a, and miR-199b that were significantly upregulated in SFN-treated H460 cells and were reported to be involved in tumorigenesis and progression (Supplementary Figure 3A & 3B).

To identify which one of the four miRNAs might mediate SFN's effect on depletion of c-MYC production, we searched miRNA binding sites by using readily available bioinformatics tools. Analyses with TargetScan, miRBase and PicTar did not predict any binding site of these miRNAs in the 3'UTR of *c-MYC* mRNA. Since recent studies have reported that miRNAs also bind to the 5'UTR and the coding sequence (CDS) of mammalian transcripts [24], we extended our search to the entire *c-MYC* mRNA sequence. Based on RNA22 and RNAhybrid algorithms, two potential miR-214 binding sites were identified at nucleotide1405 and 1683 of the *c-MYC* CDS (Figure 1B).

### *c-MYC* is an authentic target of miR-214

We next performed luciferase reporter assays to determine whether miR-214 could inhibit activity of the luciferase reporter gene by binding to the predicted target sites. For this purpose, a luciferase reporter vector psiCHECK2 containing the full length *c-MYC* CDS was constructed (named psi-*c-MYC*-CDS) and co-transfected into 293T cells together with a negative control (NC) irrelevant miRNA-mimic, miR-214-mimic, or miR-214 inhibitor. Compared with control group, miR-214 mimic markedly suppressed the luciferase activity of psi-*c-MYC*-CDS whereas miR-214 inhibitor upregulated its activity. Similar results were also observed when the luciferase reporter assay was carried out in H460 cells (Figure 1C). To confirm that miR-214 directly interacts with these two binding sites, double-stranded oligonucleotides of approximate 60bp containing the wild-type or mutated binding sites were synthesized, inserted into the psiCHECK2 vector, and named psi-1405*WT*, psi-1405*Mut*, psi-1683*WT* and psi-1683*Mut*, respectively. These luciferase reporters were then individually co-transfected into 293T cells with miR-214 inhibitor followed by luciferase assays. Compared with the NC-mimic, miR-214 mimic reduced luciferase activity by 26% with psi-1405*WT* and by 51% with psi-1683*WT* whereas no alteration was observed with vector harboring either mutant binding site (Figure 1D). These results indicate that miR-214 directly binds to the two predicted target sites. Effects of miR-214 on regulation of endogenous c-MYC protein were examined in H460 cells transfected with miR-214 mimic or inhibitor. Western blot analyses showed that c-MYC protein was reduced by miR-214 mimic and elevated by miR-214 inhibitor (Figure 1E). Taken together, these results strongly suggested that *c-MYC* is a direct target of miR-214.

### SFN negatively regulates the expression of *c-MYC* through miR-214

**Figure 2:**
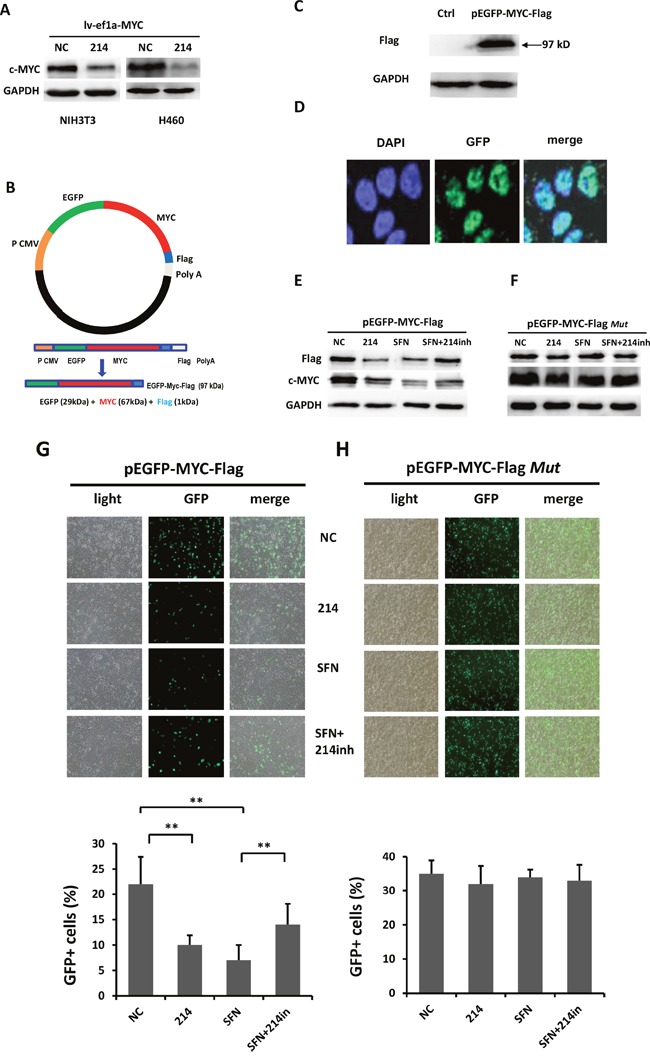
SFN negatively regulates the expression of *c-MYC* through miR-214 **A**. miR-214 suppressed exogenously expressed 3'UTR-less c-MYC in NIH3T3 and H460 cells. NIH3T3 and H460 cells were co-transfected with pLv-ef1a-c-MYC plus NC-mimic control or miR-214 mimic followed by Western blot analysis with c-MYC antibody. **B**. Construction of the pEGFP-c-MYC-Flag vector expressing a fusion protein. **C**. Western blot analysis with a Flag antibody and cell lysate derived from 293T cells transfected with pEGFP-c-MYC-Flag detected a protein band of approximate 87 kDa, the expected molecular weight of the fusion protein. **D**. 293T cells were transfected with pEGFP-c-MYC-Flag vector followed by microscopic examination of GFP green fluorescence and DAPI staining of the transfected cells. Images showed nuclear localization of the fusion protein in the cells. **E**. Either SFN or miR-214 downregulated expression of the EGFP-c- MYC-Flag fusion protein whereas inhibition of miR-214 partially rescued the suppressive effect of SFN as detected by Western blot analyses with either Flag or c-MYC antibody. **F**. Inhibitory effects of either SFN or miR-214 on the fusion protein were abolished when pEGFP-c-MYC-Flag was replaced with pEGFP-c-MYC-Flag*Mut*, which harbors the mutations of miR-214 binding sites. **G**. Fluorescence microscopic examination of EGFP expression showed that SFN or miR-214 repressed the fusion protein and the inhibitory effect of SFN was partially rescued by inhibiting miR-214. **H**. SFN and miR-214 did not affect the expression of EGFP encoded by pEGFP-c-MYC-Flag*Mut*.

Since miR-214 targets *c-MYC* potentially at its CDS instead of its 3'UTR, we examined if miR-214 represses exogenously expressed 3'UTR-less c-MYC. To this end, c-MYC was overexpressed in NIH3T3 and H460 cells by using a lentiviral vector lv-ef1a-c-MYC containing cDNA of the c-MYC CDS, but devoid of its 3'UTR. Because this vector produces abundant c-MYC protein that should constitute most majority of total c-MYC in the cells. If miR-214 targets c-MYC at its CDS, we expect to see an obvious decrease in the c-MYC level after co-transfection with lv-ef1a-c-MYC and miR-214 mimic. Western blot analysis showed that compared with NC-mimic, miR-214 mimic substantially repressed c-MYC protein levels in both NIH 3T3 and H460 cells (Figure 2A).

To further validate that *c-MYC* is an authentic target of miR-214, we subcloned the full length *c-MYC* coding region cDNA, again devoid of its 3'UTR, and a Flag epitope tag sequence into the pEGFP-C1 vector to create pEGFP-c-MYC-Flag that encodes an EGFP-c-MYC-Flag fusion protein (Figure 2B). Western blot analyses of 293T cells transfected with the recombinant plasmid revealed a protein band of approximate 87kDa, the expected size of the fusion protein consisting of EGFP (29 kDa), c-MYC (57kDa) and Flag (1 kDa) (Figure 2C), suggesting that the genetic fusion of the *EGFP-c-MYC*-*Flag* was appropriately transcribed and translated. Examination of EGFP green fluorescence and DAPI staining of the transfected cells with a confocal microscope showed nuclear localization of the fusion protein in the cells (Figure 2D). Next, we evaluated whether inhibition of miR-214 could rescue the SFN-induced repression of the c-MYC fusion protein. 293T cells were co-transfected with pEGFP-c-MYC-Flag plus NC-mimic, miR-214 mimic, or treated with SFN in the absence of presence of miR-214 inhibitor. After 48 hours, Western blot analyses were carried out. We found that either SFN or miR-214 repressed expression of the fusion protein detected by antibody against either Flag or c-MYC. Importantly, inhibition of miR-214 partially rescued the suppressive effects induced by SFN (Figure 2E). On the other hand, when pEGFP-c-MYC-Flag was replaced with pEGFP-c-MYC-Flag*Mut* that harbors mutant miR-214 binding sites, neither SFN nor miR-214 affected expression of the fusion protein (Figure 2F). Similar effects of SFN and miR-214 on the EGFP-c-MYC-Flag fusion protein were also revealed by fluorescence microscopic examination of EGFP expression (Figure 2G & 2H).

**Figure 3:**
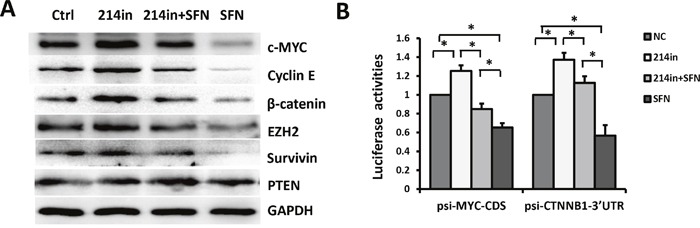
SFN/miR-214 signaling inhibits multiple oncoproteins **A**. H460 cells were treated with SFN, miR-214 inhibitor, or both of them for 48 hours. Expression levels of c-MYC, β-catenin, Cyclin E, EZH2, and Survivin were increased by miR-214 inhibitor and reduced by sulforaphane treatment. Suppressive effects of SFN were attenuated by miR-214 inhibitor. Expression of PTEN was unaffected by miR-214. **B**. SFN suppressed luciferase activities of psi-c-MYC-CDS (left panel) and psi-CTNNB1-3'UTR (right panel) in H460 cells and these effects were rescued by inhibiting miR-214. Columns, mean (n =3); bars, SD; *, *p* < 0.01.

We next examined whether inhibition of miR-214 could rescue SFN-induced downregulation of endogenous c-MYC protein. H460 cells were treated with SFN, miR-214 inhibitor, or both for 48 hours. Compared with the control group, the levels of c-MYC were increased by miR-214 inhibitor and reduced following SFN treatment. Intriguingly, the effect of SFN on repressing c-MYC was severely compromised in the presence of miR-214 inhibitor (Figure 3A). As expected, expression of Cyclin E1, a downstream target of c-MYC, was also downregulated by sulforaphane and partially rescued by miR-214 inhibitor (Figure 3A). This is in accord with our earlier EdU assay results showing S phase reduction induced by SFN (Supplementary Figure 1B). We further validated the authenticity of the SFN/miR-214/c-MYC pathway by performing luciferase reporter assays. The results showed that luciferase activity of psi-c-MYC-CDS was reduced by SFN and this effect is attenuated by miR-214 inhibitor (Figure 3B, left panel). Taken together, these results strongly suggested that SFN represses c-MYC expression through upregulating miR-214.

### SFN/miR-214 signaling downregulates multiple oncoproteins

β-catenin was reported to mediate SFN's inhibitory effect on breast cancer cells and CSCs, but the mechanisms by which SFN represses β-catenin is not elucidated [5]. Interestingly, a recent study found that the β-catenin gene *CTNNB1* is a direct target of miR-214 in human hepatocellular carcinoma [25]. Based on these observations, we presume that regulation of β-catenin by SFN is also mediated by miR-214 in NSCLC cells. To test this, luciferase reporter assay was performed to determine whether miR-214 could directly target the 3'UTR of *CTNNB1*. The results showed that luciferase activity of psiCHECK2 vector containing the target sequence of *CTNNB1*-3'UTR (psi-CTNNB1-3'UTR*WT*) was significantly decreased by miR-214 mimic and increased by miR-214 inhibitor. However, the activity of the reporter containing mutated target site (psi-CTNNB1-3'UTR*Mut*) was not affected (Supplementary Figure 4A & 4B). Similar to c-MYC, β-catenin was reduced by miR- 214 mimic and elevated by miR-214 inhibitor (Figure 1E). Further, SFN markedly repressed β-catenin protein level (Figure 3A) and activity of the luciferase reporter psi-CTNNB1-3'UTR*WT* (Figure 3B, right panel). Strikingly, these inhibitory effects of SFN were totally blocked in the presence of miR-214 inhibitor, indicating that miR-214 mediates the repressing effect of SFN on β-catenin.

It has been reported that the enhancer of zeste homologue 2 gene (*Ezh2*) was targeted by miR-214 in mouse skeletal muscle and embryonic stem cells [26] and in human hepatocellular carcinoma [25]. We found that EZH2 was upregulated following mir-214 inhibition in H460 cells, in agreement with previous reports. We further showed that EZH2 level was downregulated by SFN and this effect was partially blocked by co-treatment with miR-214 inhibitor. The oncogene *Survivin* is another target of miR-214 [27]. Similarly, we showed that Survivin was downregulated by SFN/miR-214 signaling. In contrast, expression of the tumor suppressor PTEN and p53 were not obviously affected (Figure 3A & Supplementary Figure 5).

### SFN/miR-214/c-MYC pathway regulates CSCs in NSCLC

**Figure 4:**
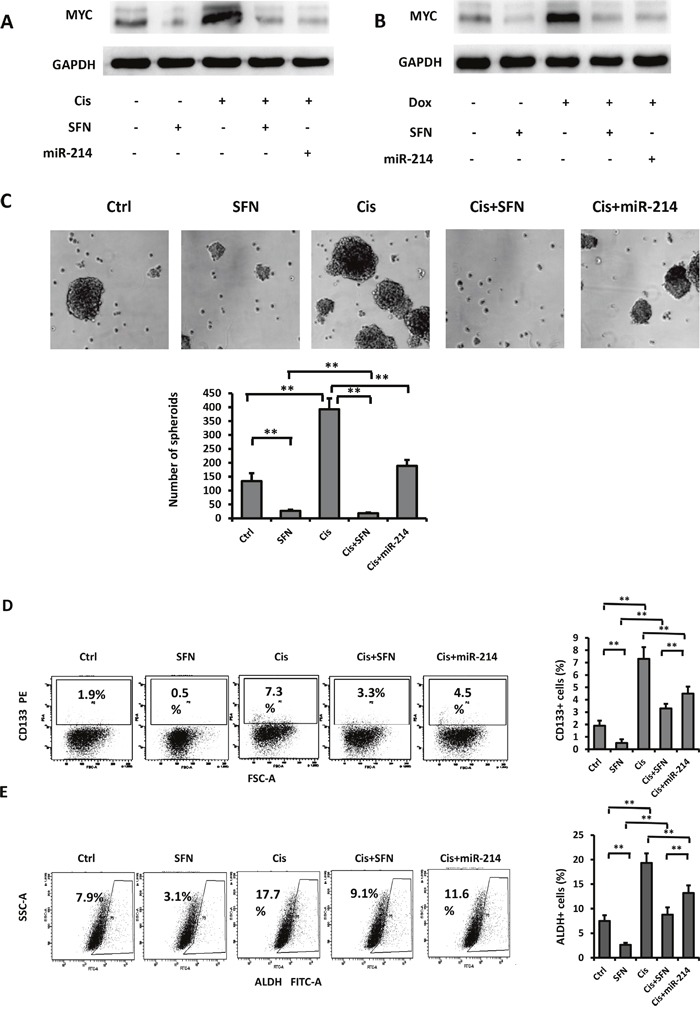
SFN/miR-214/c-MYC pathway regulates CSCs in NSCLC **A**. Treatment with cisplatin boosted c-MYC protein expression that was suppressed by either SFN or miR-214. **B**. Doxorubicin-induced c-MYC protein expression was also suppressed by SFN or miR-214. **C**. Cisplatin increases the formation of tumor spheroids, while combination of cisplatin with SFN or miR-214 decreased tumor spheroid formation. Columns, mean (n=3); bars, SD; **, *p* < 0.01. **D**. Sulforaphane or miR-214 suppressed cisplatin-enriched CD133+ population. H460 cells were treated with cisplatin (5 μmol/L), cisplatin (5 μmol/L) plus sulforaphane (10 μmol/L), or cisplatin (5 μmol/L) plus miR-214 mimic for 48 hours and subjected to flow cytometric analysis with a CD133 antibody. Columns, mean (n=3); bars, SD; **, *p* < 0.01. **E**. Aldefluor assay was carried out to detect ALDH positive cells. Sulforaphane or miR-214 suppressed cisplatin-enriched ALDH+ population. Columns, mean (n=3); bars, SD; **, *p* < 0.01.

In view of the fact that c-MYC is a crucial regulator of CSCs [13] and SFN possesses the capability in suppressing CSCs in pancreatic [4] and breast [5] cancers, we investigated functional impact of the SFN/miR-214/c-MYC pathway on CSCs in NSCLC. To this end, we firstly examined capabilities of SFN and miR-214 in modulation of c-MYC accumulation in response to cisplatin, which is a commonly prescribed chemotherapeutic drug for patients with lung cancer and is capable of inducing CSC phenotypes in NSCLCs [23]. As showing in Figure 4A, the untreated cells expressed basal level of c-MYC that was downregulated when exposed to SFN for 48 hours. In contrast, exposure of H460 cells to cisplatin strongly induced c-MYC expression. This result is in agreement with a previous report showing c-MYC level was increased by cisplatin treatment [28]. Further, cisplatin-induced c-MYC accumulation was potently suppressed by co-treatment with either SFN or miR-214. Doxorubicin is another chemotherapeutic drug for lung cancer patients. Similarly, either SFN or miR-214 repressed doxorubicin-induced c-MYC expression (Figure 4B).

We next examined the role of SFN/miR-214/c-MYC signaling in tumor spheroid formation and CD133 surface marker expression that are surrogates for CSCs of NSCLC [23, 29]. To assess tumor spheroid formation, cells were cultured in serum-free medium containing bFGF and EGF. After 10 days of cultivation, floating tumor spheroids were observed in control samples. While SFN abolished spheroid formation, cisplatin markedly increased the number of the spheroids (Figure 4C). However, the effect of cisplatin was totally blocked by SFN and partially reversed by miR-214. Next, flow cytometric analysis with a CD133 antibody showed that CD133+ population was enriched by cisplatin and this effect was partially blocked by either SFN or miR-214 (Figure 4D). Aldehyde dehydrogenase (ALDH) is another CSC marker of NSCLC [30, 31], ALDH positive cells are highly tumorigenic, clonogenic and capable of self-renewal. We performed Aldefluor assay to measure ALDH+ cell population. Similar to CD133+, ALDH+ population enriched by cisplatin was reduced by either SFN or miR-214.

### SFN increases chemotherapeutic drug-induced toxicity in H460 cells

**Figure 5:**
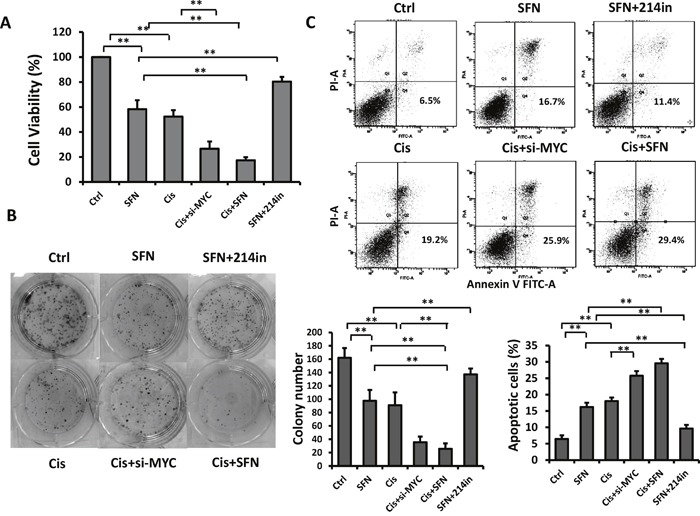
SFN increases chemotherapeutic drug-induced toxicity in H460 cells **A**. SFN potentiated cisplatin's inhibitory effect on H460 cells as measured by using MTT assay. Cells were treated with SFN (10μM), cisplatin (5 μM), c-MYC siRNA, or their combination for 72 hours. Columns, mean (n =3); bars, SD; *, p < 0.05; **, p < 0.01. **B**. H460 cells were plated at low density in 6-well plates, and then cells were treated with SFN, cisplatin, c-MYC siRNA, or their combination. After 15 days, colonies were stained with coomassie blue and images of colonies were taken. Columns, mean (n =3); bars, SD. **, p < 0.01. **C**. SFN enhanced cisplatin's effect on apoptosis induction as evidenced by flow cytometric analysis with propidium iodide and Annexin V double staining. Columns, mean (n =3); bars, SD. *, *p* < 0.05; **, p < 0.01.

Having demonstrated the potent inhibitory effect of SFN/mR-214 on c-MYC and giving the fact that c-MYC plays critical roles in chemotherapy resistance in various tumor types [28, 32] and depletion of *c-MYC* with antisense RNA or RNAi restored sensitivities in tumor cells [33, 34], we reasoned that SFN could increase cell sensitivity to chemotherapeutic drug-mediated cytotoxicity. As shown in Figure 5A, treatment of H460 cells with SFN or cisplatin reduced cell viability by 41% or 45%, respectively, whereas combination of SFN with cisplatin was more effective than either reagent used alone and reduced cell viability by 80%. Importantly, repressive effect of SFN on cell viability was substantially compromised in the presence of miR-214 inhibitor, suggesting that function of SFN is mediated by miR-214. Further, we found that knockdown of c-MYC with siRNA enhanced inhibitory effect of cisplatin, in agreement with previous observations that c-MYC depletion potentiates cisplatin sensitivity. However, this combination (Cis plus *c-MYC* siRNA) was less effective than cisplatin plus SFN, suggesting that SFN exerts its functions not only by suppressing c-MYC, but also through other signaling pathways such as those we demonstrated in Figure 3A. Likewise, combined treatment with SFN and cisplatin reduced colony formation number significantly compared to a single reagent used alone. The effect of SFN was attenuated by miR-214 inhibitor while cisplatin sensitivity was enhanced by *c-MYC* siRNA (Figure 5B). The ability of combined treatment to induce apoptosis was assessed by using flow cytometric analysis with propidium iodide and Annexin V double staining. The results showed that SFN plus cisplatin markedly increased apoptosis when compared to single treatments. Apoptosis-inducing effect of SFN was diminished by miR-214 inhibitor while the effect of cisplatin was enhanced by *c-MYC* siRNA, respectively (Figure 5C). Similarly, SFN also repressed doxorubicin-induced c-MYC expression (Figure 4B) and enhanced its cytotoxicity (Supplementary Figure 6).

### SFN sensitizes H460 cells to chemotherapy *in vivo*

**Figure 6:**
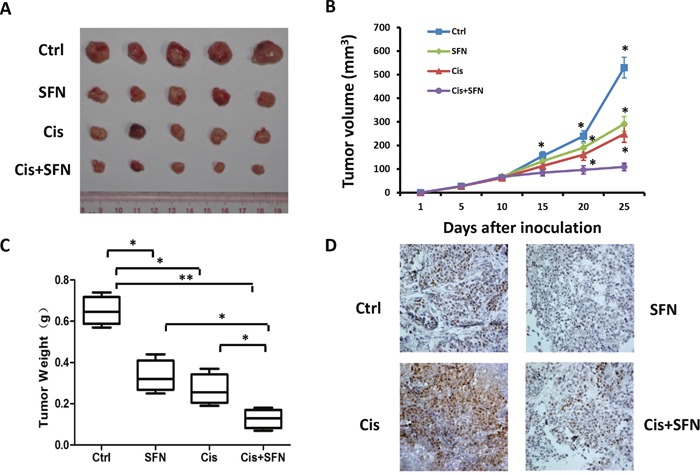
SFN potentiated cisplatin-mediated efficacy *in vivo* **A**. Subcutaneous xenografts of H460 cells in nude mice were treated with vehicle, SFN, cisplatin, or SFN plus cisplatin. **B**. Tumor volume (mm^3^) was measured at the indicated treatment days 5, 10, 15, 20 and 25. **C**. Tumor weights were determined after the treatment was completed at day 25. Data represent the mean ± SD; n=5.*, *p* < 0.05; **, p < 0.01. **D**. Immunohistochemical staining of the primary tumors with an anti-c-MYC antibody. SFN inhibited the basal level and cisplatin-induced accumulation of c-MYC protein (magnification, ×40).

To further investigate whether SFN could potentiate NSCLC cell sensitivity toward chemotherapy *in vivo*, we performed experiments in xenograft model with nude mice. H460 cells were implanted into flanks of mice by subcutaneous injection. Mice were intraperitoneally injected with vehicle, SFN, cisplatin or both agents together. Compared to control group, administration of SFN or cisplatin alone inhibited tumor growth and tumor volumes was markedly reduced by 55% or 45%, respectively. Combination treatment with SFN and cisplatin further enhanced the anti-tumor effect compared to each single treatment (Figure 6A, 6B and 6C). To determine the *in vivo* effect of SFN on c-MYC expression, tumor sections were subjected to immunohistochemical analyses with anti-c-MYC antibody. While cisplatin induced c-MYC accumulation, SFN effectively inhibited expression levels of c-MYC either in the absence or in the presence of cisplatin (Figure 6D). These results suggested that SFN potentiates cisplatin-induced cytotoxicity at least partly through inhibiting c-MYC expression. In summary, our *in vitro* and *in vivo* experiments demonstrated that SFN inhibited basal level or cisplatin-stimulated c-MYC expression in lung cancer cells, which are accompanied by enhanced therapeutic efficacy.

## DISCUSSION

c-MYC modulates a wide range of fundamental biological processes and plays a pivotal role in tumor initiation and progression. Deregulated c-MYC expression is detected in most types of cancer including NSCLC and is often associated with aggressive, poorly differentiated tumors [35], which make c-MYC an attractive anti-cancer target. Though therapeutic approaches aimed at targeting c-MYC are still not available in clinic, remarkable progress in switching c-MYC off have been made by researchers around the world. Recent studies found that systemic suppression of endogenous Myc with dominant-negative Myc mutant Omomyc triggered rapid and complete regression of KRas-driven lung adenomas with only mild and fully reversible side effects [36]. Inhibition of endogenous Myc with Omomyc also triggers ubiquitous regression of tumors in a simian virus driven pancreatic b-cell mouse tumor model [37]. These data support the notion that inhibiting c-MYC is a promising strategy for treating diverse malignancies. In the present study, we provided strong evidences demonstrating that SFN, a natural compound derived from broccoli and other cruciferous vegetables, upregulated miR-214 that, in turn, repressed *c-MYC* by binding to its CDS. This finding was substantiated by our observations that plasmid or lentiviral vector-mediated expression of 3'UTR-less *c-MYC* cDNA and chemotherapeutic drug cisplatin- or doxorubicin-induced endogenous c-MYC accumulation was also suppressed by either SFN or miR-214. SFN acts as a potent chemopreventive agent against cancer through modulating multiple signaling pathways involved in cell growth, apoptosis, inflammation and self-renewal of CSCs [4, 5, 38, 39]. Our study provides key mechanistic insights into how sulforaphane inhibits c-MYC activity, thereby exerting its anti-cancer and CSC effects. Since SFN treatment has shown to be safe and well tolerated in clinic trials, using SFN as a c-MYC inhibitor may have significant impact on effective therapeutic strategy for patient with c-MYC-related tumors.

CSCs are intrinsically resistant to chemotherapy and radiation therapy [40–42], therefore targeting CSCs may provide an effective anticancer strategy to overcome resistance and reduce recurrence. In view of recent finding that c-MYC is a crucial regulator of CSCs [13] and chemoresistance [28] in various malignancies, we examined the biological consequences of SFN/miR-214-mediated inhibition c-MYC in NSCLC. Our results showed that treatment with either cisplatin or doxorubicin stimulated c-MYC accumulation that was effectively repressed by co-treatment with SFN or by ectopic expression of miR-214. SFN/miR-214 inhibited CSC properties and enhanced chemotherapeutic drug-induced cytotoxicity. Importantly, SFN sensitized NSCLC cells to cisplatin's efficacy *in vivo*, which is associated with inhibition of cisplatin-induced c-MYC accumulation in tumor tissues.

SFN has been shown to possess anti-CSC and anti-tumor effect through inhibiting β-catenin in breast cancer cells [5]. However, the mechanism by which SFN regulates β-catenin has not been elucidated. Here we demonstrated that similar to c-MYC, β-catenin is also regulated by SFN via miR-214 in NSCLC cells. Since c-MYC is a known target of β-catenin [43], thus in addition to direct regulation by miR-214, c-MYC can be indirectly regulated by miR-214 through β-catenin. A recent study with transgenic β-catenin mouse models found that constitutive activation of β-catenin signaling in the mammary basal cell layer induces tumors similar to that of basal-like human breast carcinomas. However, conditional deletion of *Myc* from the transgenic mice abolished the regenerative capacity of basal epithelial cells and completely prevented the tumorigenesis. Thus, Myc pathway activation was considered to be essential for β-catenin-induced stem cell amplification and tumorigenesis [44]. In this regard, direct and indirect inhibition of c-MYC can be considered as a crucial function of miR-214 as a tumor suppressing regulator in NSCLC cells.

In conclusion, we demonstrated that SFN inhibited CSC properties and enhanced therapeutic efficacy of cisplatin in NSCLC. SFN exerted these functions through upregulation of miR-214 expression, which in turn targeted c-MYC, β-cantenin, and EZH2. Our results provided strong evidence and mechanisms to support consideration of SFN as a therapeutic agent in combination with cisplatin for the treatment of patients with NSCLC and, potentially, other types of tumors.

## MATERIALS AND METHODS

### Cell culture, transfection and reagents

Human normal lung bronchial epithelium cell line BEAS-2B and NSCLC cell line H460, H1299 and A549 were obtained from American Type Culture Collection (Manassas, VA) and were routinely tested for absence of mycoplasma using a Mycoplasma Detection Kit (Bitool, China) in our laboratory. All cell lines were maintained in DMEM supplemented with 10% fetal bovine serum. bFGF, EGF and B27 were purchased from Invitrogen (Carlsbad, CA). SFN was from LKT Laboratories. Cisplatin, Hoechst33342 and propidium iodide were from Sigma-Aldrich (St Louis, MO). Antibodies against c-MYC, Cyclin E, EZH2, Survivin, and p53 were acquired from Abcam (Cambridge, UK). Caspase 3 and cleaved caspase 3 were from Cell Signaling, Inc (Danvers, MA). β-catenin and PTEN were from Santa Cruz Biotechnology, Inc (Santa Cruz, CA). miRNAs and vectors were transfected using Lipofectamine 2000 reagent (Invitrogen). The miR-214 mimic, negative control, and miR-214 inhibitor were purchased from GenePharma (Suzhou, China).

### Cell viability assay

Cells were seeded in 96-well microplates at a density of 5,000 cells per well and incubated overnight. After treatment for 48 hours, cell viability was assessed by MTT assay (Sigma-Aldrich). Absorbance was measured at 590 nm using a microplate reader. Cell survival was expressed as absorbance relative to that of untreated controls.

### EdU assay

Cells were incubated with 50 μM of 5-ethynyl-2'-deoxyuridine (EdU, RiboBio, Guangzhou, China) at 37°C for 2 hours. Then, cells were fixed in 4% paraformaldehyde. After permeabilization with 0.5% Triton-X, cells were reacted with reaction cocktail for 30 min. Subsequently, cell nucleus were stained with Hoechst 33342 for 30 min and visualized under a Nikon Eclipse TE2000-S microscope (Nikon Imaging, Japan) and the photos were acquired with a Nikon Ti-U microscope (Nikon Imaging, Japan).

### Apoptosis assay

Cells were grown in 10-cm dishes and treated with SFN (10μmol/L). After 48 hours incubation, cells were trypsinized, washed twice with cold PBS, and resuspended in 500μl Annexin V-FITC binding buffer (1×10^7^cell/mL). Next, 5μl Annexin V FITC and 100μl PI were added into each sample. Cells were kept in a dark place at room temperature for 15 minutes before the proportion of apoptosis cells were analyzed using a FACSAria Flow cytometer (Beckton Dickson, San Jose, CA).

### Tumor spheroid formation assay

Cells were plated in six-well ultralow attachment plates (Corning Inc., Corning, NY) and grown in a serum-free mammary epithelium basal medium (Lonza Inc, Allendale, NJ) supplemented with 100 U/mL penicillin and 100 U/mL streptomycin, 20 ng/mL EGF (Sigma-Aldrich), 20 ng/mL bFGF (Sigma-Aldrich), and B27 (Invitrogen). SFN (5 μmol/L) was added to the tumor spheroid culture. After 10 days of culture, the number of tumor spheroids was counted under a Nikon Eclipse TE2000-S microscope (Nikon Imaging, Japan) and the photos were acquired with a NikonTi-U microscope (Nikon Imaging, Japan).

### CD133+ cells analysis

After treatment with SFN and/or cisplatin, cells were detached from the dishes with Trypsin-EDTA (Invitrogen) and suspended at 1×10^5^ cells/mL in PBS supplemented with 2% fetal calf serum. These cells were then incubated with an anti-CD133 antibody (Miltenyi Biotec, Bergisch Gladbach, Germany) in a refrigerator (4°C) for 10 minutes. After washing, CD133-positive cells were detected by using a FACSAria Flow cytometer (BD Biosciences, San Jose, CA).

### Aldefluor assay and cell sorting

The Aldefluor kit (Stem Cell Technologies, Vancouver, Canada) was used to isolate an Aldefluor-stained cell population with ALDH1 enzymatic activity [45]. The experiments were performed according to the manufacturer's instruction. Briefly, cells were suspended in Aldefluor assay buffer containing ALDH1-substrate, BODIPY-aminoacetaldehyde (BAAA), and incubated for 45 min at 37°C. BAAA was converted by intracellular ALDH into a fluorescent product BODIPY-aminoacetate, which was detected by using a FACSAria Flow cytometer.

### Western blotting analysis

After different treatment, cells were lysed in NP-40 lysis buffer supplemented with a protease inhibitor cocktail (Pierce Biotechnology/Thermo Fisher Scientific, Waltham, MA). Cell lysate was centrifuged at 14,000 rpm for 15 minutes and the supernatant was recovered. Protein concentration was determined with BCA Protein Assay Reagents (Pierce). Equal amounts of protein were subject to SDS-PAGE and transferred to a polyvinylidene difluoride membrane (Millipore, Billerica, MA). The membrane was then incubated with appropriate antibodies. Protein bands were detected by enhanced chemiluminescence reagents according to manufacturer's instructions.

### RNA isolation, reverse transcription, and quantitative real-time PCR

Total RNAs were extracted using Trizol reagent (Invitrogen). Total RNAs were polyadenylated and reversely transcribed using NCode miRNA First-Strand cDNA Synthesis Kit (Invitrogen). Quantitative real-time PCR (qPCR) was performed using SYBR Green PCR master mix (Applied Biosystems, Waltham, MA) on an Mx3005P system (Stratagene, La Jolla, CA). The primers were listed in Supplementary Table. U6 was used as an endogenous control. All samples were normalized to internal controls and fold changes were calculated through relative quantification (2-ΔΔCt).

### Construction of various vectors

To construct the mammalian expression plasmid pEGFP-c-MYC-Flag, cDNA of the full-length *c-MYC* coding region was in frame inserted down-stream of the EGFP gene in the pEGFP-C1 plasmid (Clontech, Mountain View, CA). In addition, a double strand oligonucleotide encoding the Flag epitope tag was chemically synthesized and in frame cloned downstream of the *c-MYC* CDS. The resulted recombinant vector contains genetic fusions of the EGFP, *c-MYC*, and Flag tag. The pEGFP-c-MYC-Flag mutant constructs were made by PCR using a QuikChange site-directed mutagenesis kit (Stratagene). The oligonucleotide primers were designed to introduce the mutations of miR-214 target sites without affecting amino acid sequences. The psi-c-MYC-CDS luciferase reporter vector was created by insertion of the full-length *c-MYC* coding region into the Xho I and Pme I sites of the psiCHECK-2 dual luciferase reporter (Promega, Madison, WI). To generate the psi-1405*WT*, psi-1405*Mut*, psi-1683*WT* and psi-1683*Mut* luciferase reporters, double-strand oligonucleotides containing the wild-type or mutant miR-214 binding sites were synthesized and cloned into the Xho I and Pme I sites of the psiCHECK-2 dual luciferase reporter. The wild or mutated full-length β-catenin gene *CTNNB1* 3'UTR was cloned into the PmeI and Not I sites of the psiCHECK-2 luciferase reporter. The sequences of oligonucleotides were listed in Supplementary Table.

### Luciferase assay

Cells were co-transfected with a luciferase reporter vector along with miRNA mimic or inhibitor. The luciferase signals were measured 24 hours after transfection, using the Dual-Glo Luciferase Assay System (Promega, Madison, WI) and a Panomics luminometer (Panomics, Fremont, CA) according to the manufacturers' instructions.

### Colony-forming assay

Tumor cells were seeded at a density of 500 cells per wells in 6-well tissue culture plates. Twenty-four hours later, cells were treated differently for 14 days. For determination of colony formation, cultures were fixed with 4% paraformaldehyde and stained with hematoxylin. The number of colonies with >50 cells was counted under a dissecting microscope.

### Nude mice and tumor xenografts

Animal studies were conducted in strict accordance with the principles and procedures approved by the Committee on the Ethics of Animal Experiments of Southern Medical University. Nude mice (BALB/C nu/nu) were fed autoclaved water and laboratory rodent chow. H460 cells (2×10^6^ in 150μl) were transplanted into right flanks of nude mice by subcutaneous injection (day 0). Seven days after implantation, mice carrying tumor xenografts were randomly separated into four groups (5 mice per group). Then mice were intraperitoneally injected with vehicle, SFN (4mg/kg), cisplatin (3mg/kg) or SFN (4mg/kg) plus cisplatin (3mg/kg) every 3 days for a total of 6 doses. Tumor volumes (V) were calculated using the formula V=1/2 (length x width^2^). Mice were euthanized when the mean tumor volume in the control group reached 500mm^3^. Tumor tissues were collected, fixed in 10% formaldehyde, and embedded in paraffin for immunohistochemical analysis of c-MYC expression.

### Statistical analysis

All values are expressed as means ± standard error. Results were analyzed by one-way analysis of variance or t-test as appropriate with the program SPSS13.0. Differences between two means with *p* < 0.05 were considered significant.

## SUPPLEMENTARY MATERIALS FIGURES AND TABLES




