# Supplementary material for: Sulforaphane inhibits cancer stem-like cell properties and cisplatin resistance through miR-214-mediated downregulation of c-MYC in non-small cell lung cancer
Source: Oncotarget. 2017 Jan 5;8(7):12067–80. doi: 10.18632/oncotarget.14512 (PMC5355326; doi:10.18632/oncotarget.14512)
Supplement: Supplementary file 1 [file oncotarget-08-12067-s001.pdf]

Sulforaphane inhibits cancer stem-like cell properties and cisplatin resistance through miR-214-mediated downregulation of c-MYC in non-small cell lung cancer

SUPPLEMENTARY FIGURES AND TABLE

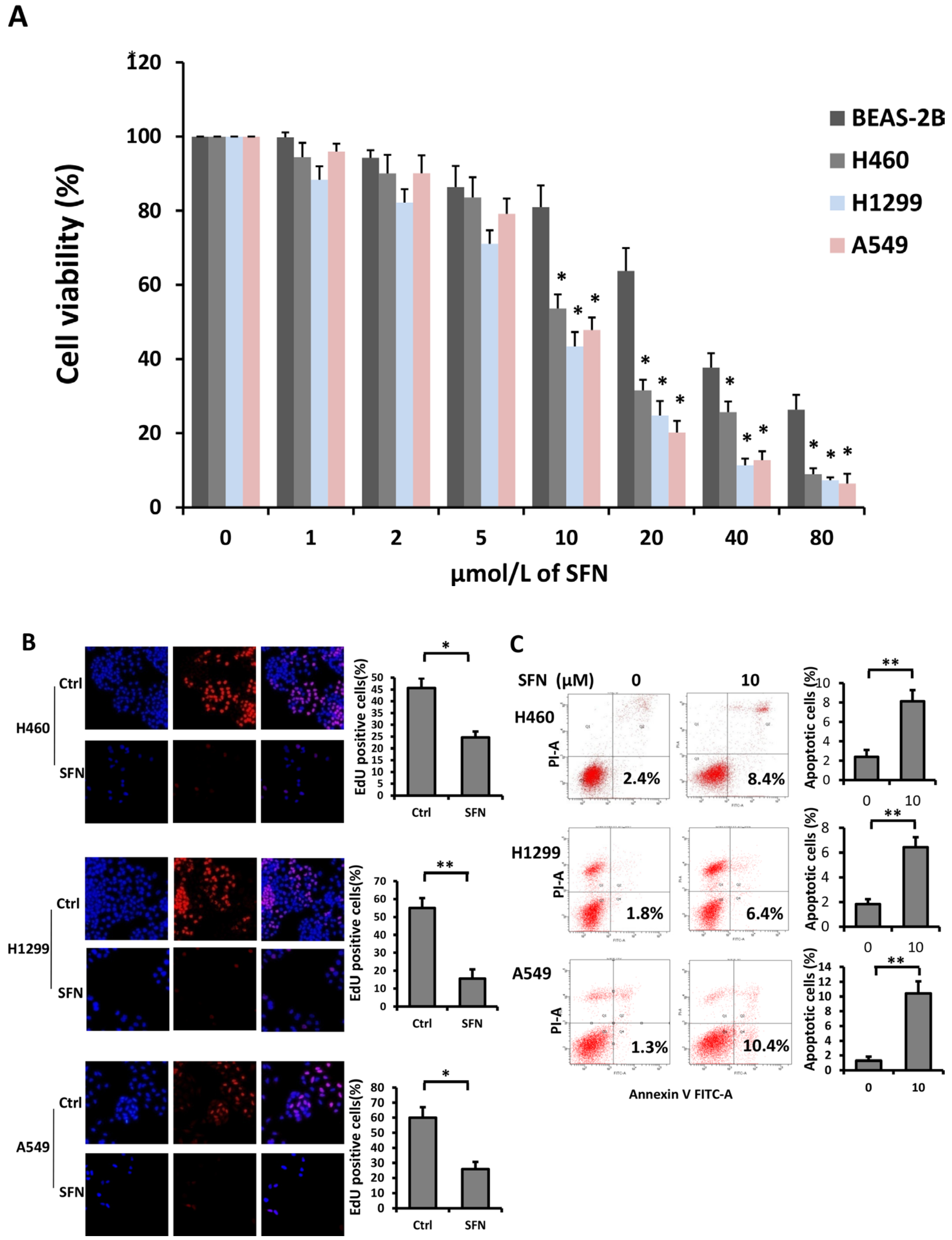

Supplementary Figure 1: SFN inhibits viability and induces apoptosis of NSCLC cells. Cells growing in log phase were treated with increasing concentrations of SFN for 48 hours followed by various cellular assays. **A.** SFN suppressed cell viability as measured by using MTT assay. Columns, mean (n =3); bars, SD; *, $p< 0.05$. **B.** Treatment with SFN (10 µmol/L) for 48 hours resulted in S phase reduction determined by the EdU incorporation assay. **C.** Treatment with SFN (10 µmol/L) for 48 hours induced apoptosis as assessed by Annexin V staining. Columns, mean (n =3); bars, SD; *, $p < 0.05$; **, $p< 0.01$.

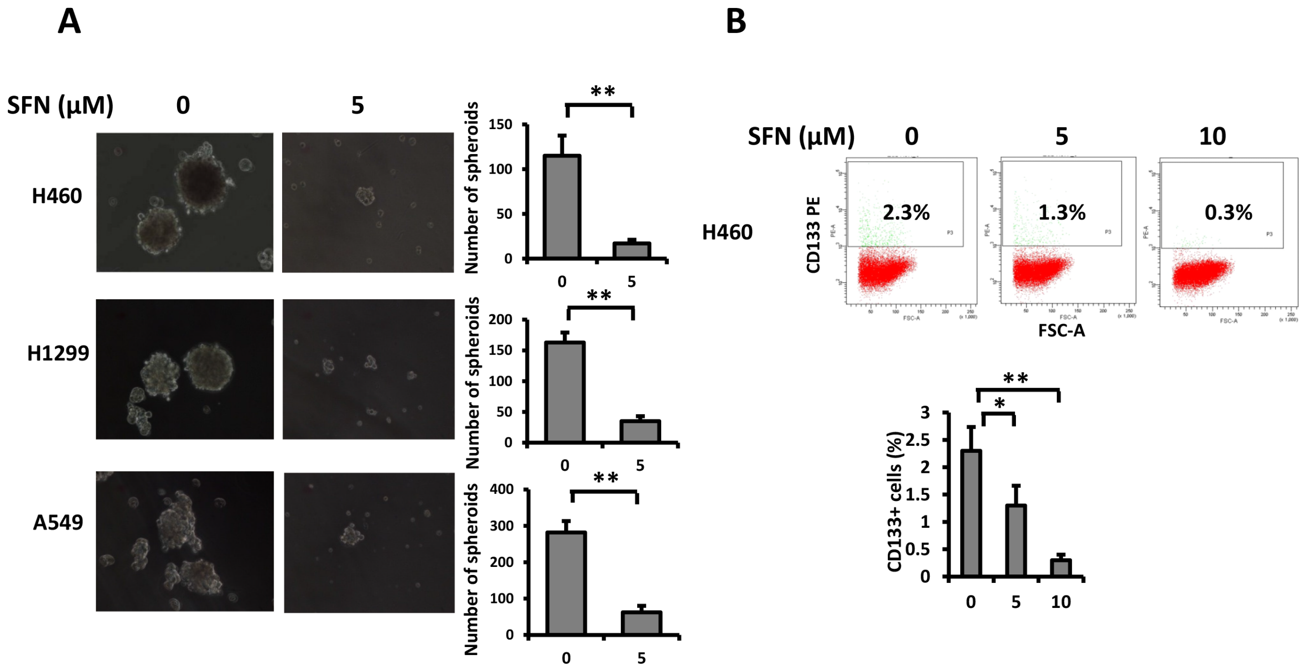

Supplementary Figure 2: SFN inhibits CSC properties in NSCLC cells. A. SFN decreased tumor spheroid formation in NSCLC cells. H460, H1299 and A549 cells were cultured in serum-free medium containing EGF and bFGF. Treatment with SFN (5 μmol/L) for 10 days reduced the number of tumor spheroids. Columns, mean (n =3); bars, SD;**, $p < 0.01$. **B.** SFN decreased CD133+ cells. H460 cells were treated with SFN (5 or 10 μmol/L) for 48 hours and subjected to flow cytometric analysis with a CD133 antibody. Columns, mean (n =3); bars, SD; *, $p < 0.05$; **, $p < 0.01$.

A

microRNA	Expression in sulforaphane-treated H460 cells	Chrom
miR-101	up	1p13
miR-122	up	18q21
miR-145	up	5q32-33
miR-185	up	22q11
miR-199a	up	19p13/1q24
miR-199b	up	9q34
miR-214	up	1q23
miR-124	down	8q12.3
miR-302b	down	4q13

B

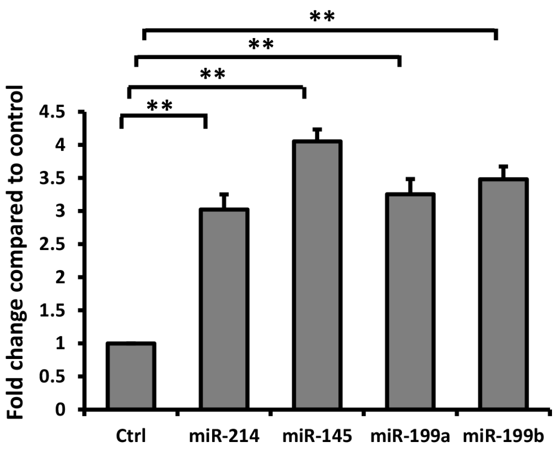

Supplementary Figure 3: Identification of SFN-modulated miRNAs in H460 cells. A. miRNAs differentially expressed between the control and SFN-treated H460 cells. **B.** Relative expression levels of each miRNA were quantitated with TaqMan real-time PCR microRNA assays. Columns, mean (n =3); bars, SD; **, $p < 0.01$

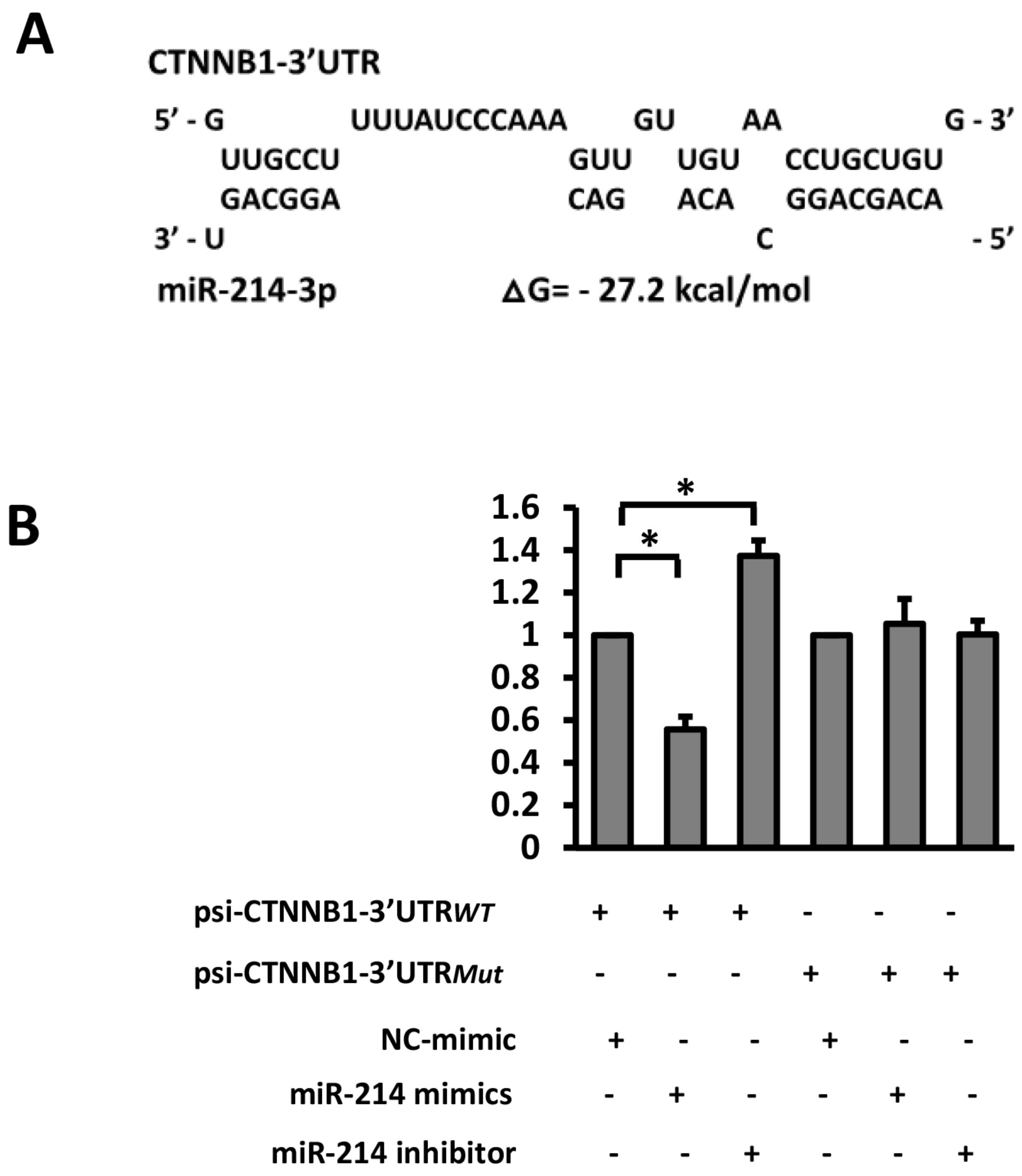

Supplementary Figure 4: miR-214 negatively regulates β-catenin through targeting its 3'UTR. **A.** Sequence alignment of miR-214 and 3'UTR of CTNNB1. **B.** H460 cells were co-transfected with NC-mimic control, miR-214 mimic or miR-214 inhibitor plus a luciferase reporter containing the full length of β-catenin 3'UTR (psi-β-catenin-3'UTR*WT*) or a mutated binding site (psi-β-catenin-3'UTR*Mut*). Luciferase activities of psi-β-catenin-3'UTR*WT* was suppressed with miR-214 mimic and elevated with miR-214 inhibitor whereas activities of psi-β-catenin-3'UTR*Mut* was unaffected. Columns, mean (n =3); bars, SD; *, p <0.05.

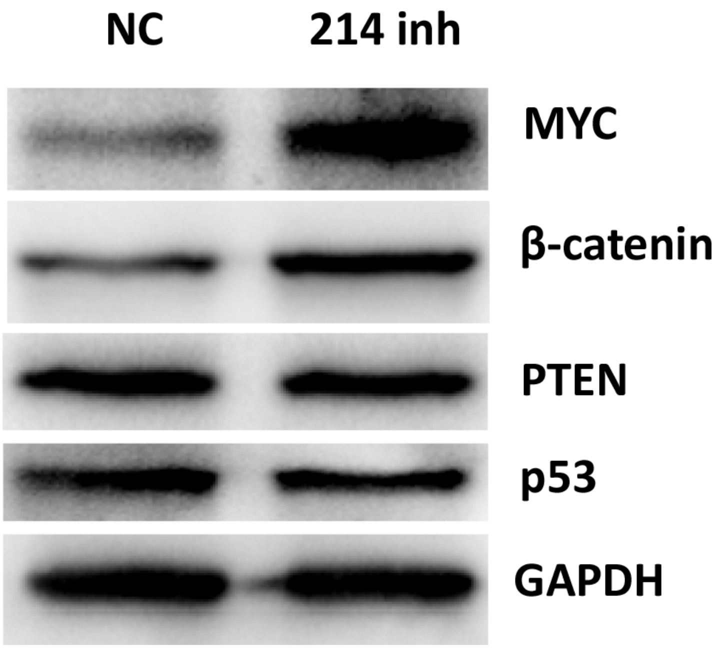

Supplementary Figure 5: Inhibition of miR-214 resulted in upregulation of MYC and β-catenin while PTEN and p53 were unaffected. H460 cells were treated without or with miR-214 inhibitor for 48 hours. Expression levels of c-MYC, β-catenin, PTEN, and p53 were examined.

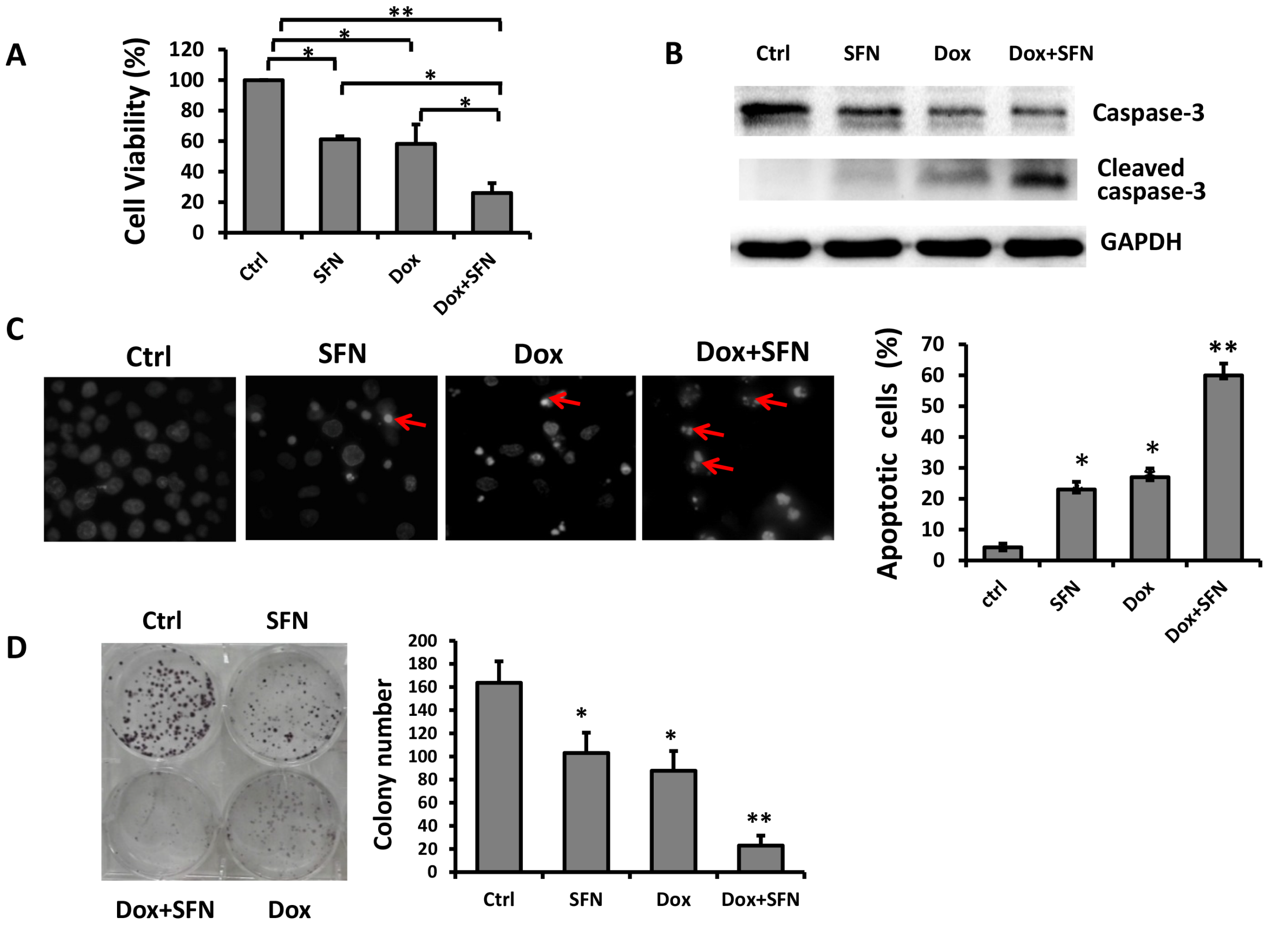

Supplementary Figure 6: Sulforaphane increases doxorubicin-induced toxicity in H460 cells. A. SFN potentiated doxorubicin's inhibitory effect on H460 cells as measured by using MTT assay. Cells were treated with SFN (10µM), doxorubicin (3 µM), or both for 72 hours. Columns, mean (n =3); bars, SD; *, $p < 0.05$; **, $p < 0.01$. **B&C.** SFN enhanced doxorubicin's effect on apoptosis induction as evidenced by Western blot analysis with an anti-Caspase 3 antibody (B) and by Hochest 33342 staining (C). Columns, mean (n =3); bars, SD. *, $p < 0.05$; **, $p < 0.01$. **D.** H460 cells were plated at low density in 6-well plates, and then cells were treated with SFN, doxorubicin, or SFN combined with doxorubicin. After 15 days, colonies were stained with coomassie blue and images of colonies were taken. Columns, mean (n =3); bars, SD; *, $p < 0.05$; **, $p < 0.01$.

Supplementary Table: Left panel: Primers used in quantitative real-time PCR experiments. Right panel: Oligonucleotides used in vector construction

See Supplementary File 1
