# Supplementary material for: Sulforaphane inhibits cancer stem-like cell properties and cisplatin resistance through miR-214-mediated downregulation of c-MYC in non-small cell lung cancer
Source: Oncotarget. 2017 Jan 5;8(7):12067–80. doi: 10.18632/oncotarget.14512 (PMC5355326; doi:10.18632/oncotarget.14512)
Supplement: Supplementary file 2 [file oncotarget-08-12067-s002.docx]

**Primers used in quantitative real-time PCR experiments**

|  | **Primers** | **Sequences** |
| --- | --- | --- |
| **miR-214** | **RT primer** | **CTCAACTGGTGTCGTGGAGTCGGCAATTCAGTTGAGACTGCCTGTCTGTGCCTGCTGT** |
|  | **Forward** | **ACACTCCAGCTGGGACAGCAGGCACAGACAGGCAGT** |
| **miR-145** | **RT primer** | **CTCAACTGGTGTCGTGGAGTCGGCAATTCAGTTGAGAGGGATTCCTGGGAAAACTGGAC** |
|  | **Forward** | **ACACTCCAGCTGGGGTCCAGTTTTCCCAGGAATCCCT** |
| **miR-199a** | **RT primer** | **CTCAACTGGTGTCGTGGAGTCGGCAATTCAGTTGAGGAACAGGTAGTCTGAACACTGGG** |
|  | **Forward** | **ACACTCCAGCTGGGCCCAGTGTTCAGACTACCTGTTC** |
| **miR-199b** | **RT primer** | **CTCAACTGGTGTCGTGGAGTCGGCAATTCAGTTGAGTAACCAATGTGCAGACTACTGT** |
|  | **Forward** | **ACACTCCAGCTGGGACAGTAGTCTGCACATTGGTTA** |
| **miRNAs** | **Reverse** | **TGGTGTCGTGGAGTCG** |
| **U6** | **Forward** | **CTCGCTTCGGCAGCACA** |
|  | **Reverse** | **AACGCTTCACGAATTTGCGT** |

**Oligonucleotides used in vector construction**

|  | **Primers** | **Sequences** |
| --- | --- | --- |
| **psi-MYC-CDS** | **Forward** | **CCGCTCGAGATGCCCCTCAACGTTAGCTTC** |
|  | **Reverse** | **AGCGTTTAAACCGCACAAGAGTTCCGTAGCTG** |
| **psi-CTNNB1-3’UTR*WT*** | **Forward** | **AGCTGTTTAAACACTCTGCCTACAGAACTTCAG** |
|  | **Reverse** | **ATAGTGCGGCCGCGAAGCATCGTATCACAGCAGG** |
| **psi-CTNNB1-3’UTR*Mut*** | **Forward** | **CTCAACTGGTGTCGTGGAGTCGGCAATTCAGTTGAGGAACAGGTAGTCTGAACACTGGG** |
|  | **Reverse** | **ACACTCCAGCTGGGCCCAGTGTTCAGACTACCTGTTC** |
| **pEGFP-MYC-Flag** | **Forward** | **GGAAGATCTCCCCTCAACGTTAGCTTCAC** |
|  | **Reverse** | **CCCAAGCTTCTTATCGTCGTCATCCTTGTAATCCATCGCACAAGAGTTCCGTAGCT** |
| **pEGFP-MYC-Flag-*Mut*** | **1405-1** | **GAGTCTGGATCACCAAGCGCAGGAGGCCACAGCAAAC** |
|  | **1405-2** | **GTTTGCTGTGGCCTCCTGCGCTTGGTGATCCAGACTC** |
|  | **1683-1** | **CGGAGCTTTTTTGCATTAAGAGACCAGATCCCG** |
|  | **1683-2** | **CGGGATCTGGTCTCTTAATGCAAAAAAGCTCCG** |

|  | **sequences** |
| --- | --- |
| ***c-Myc* siRNA** | **AACGUUAGCUUCACCAACAUU** |
| **control siRNA** | **AATTCTCCGAACGTGTCACGT** |
